# Habitual behavior and dopamine cell vulnerability in Parkinson disease

**DOI:** 10.3389/fnana.2015.00099

**Published:** 2015-08-06

**Authors:** Ledia F. Hernández, Peter Redgrave, José A. Obeso

**Affiliations:** ^1^Fundacion HM, Hospital HM Puerta del Sur, Centre for Integrative Neuroscience A.C., Mostoles and CEU San Pablo UniversityMadrid, Spain; ^2^Center for Networked Biomedical Research on Neurodegenerative Diseases, Institute Carlos IIIMadrid, Spain; ^3^Department of Psychology, University of SheffieldSheffield, UK

**Keywords:** Parkinson disease, dopamine, substantia nigra, habits, goal directed behavior, vulnerability factors, striatum

The cardinal features of Parkinson disease (PD) often begin focally, typically in one limb, and may remain relatively restricted to one side of the body for many years. It is now well established that dopaminergic neurons in the ventro-lateral tier of the substantia nigra pars compacta (SNpc), which project mainly to the caudal putamen, are the first to degenerate in the initial phase of PD (Fearnley and Lees, 1991; Halliday et al., 2008; Blesa et al., 2010) indicating differential vulnerability. The caudal region of the striatum (dorsolateral striatum in rodents) has been associated with habitual (or automatic) behavior (Redgrave et al., 2010), consequently the differential loss of dopamine (DA) from this region provides the pathophysiological substrate for the early impairment of automatic movements (walking, writing, …) in early PD.

The brain has two major systems for controlling behavior: a goal directed mechanism (GD) and a mechanism mediating stimulus-response habits (Figure 1). The goal directed system entails conscious, voluntary control of actions aimed toward obtaining rewards or avoiding punishments. Action selection is determined by competitions between relative outcome values, i.e. if outcome A is more valuable than outcome B, then learned behavior that will lead to outcome A will be selected. Examples of goal-directed control would be: heading to the fridge or going to a restaurant when we are hungry, taking the elevator or taking the stairs back to the apartment. This goal-directed process engages the prefrontal cortex and dorsolateral striatum (Yin et al., 2004). On the other hand, the habitual system detects well-learned cues that have been associated with specific responses, and therefore elicit automatic stimulus-response behavior via the re-entrant loop that connect sensorimotor cortical areas with the posterior putamen (dorsolateral striatum in rodents) (Barnes et al., 2005). Habits are established gradually over time. They evolve after many repetitions of a task being performed under flexible goal-directed learning and depend heavily on the statistical regularities between specific stimuli and consequent responses. Examples of habitual control would be, walking, riding a bike or driving. The critical test for habits is that they are resistant to outcome-devaluation (Adams and Dickinson, 1981). Inappropriate habitual responses are frequently difficult to eradicate and have to be corrected by goal-directed interventions after they fail to achieve their original intention. In this Opinion article we put forward the hypothesis that a significant factor that confers vulnerability to the ventro-lateral tier of SNpc at the onset of PD may reside in the key functional role that these neurons play in the performance of habitual behavior, switching between habitual and goal-directed control, and engaging both goal-directed and habitual control when carrying out multiple tasks simultaneously.

**Figure 1:**
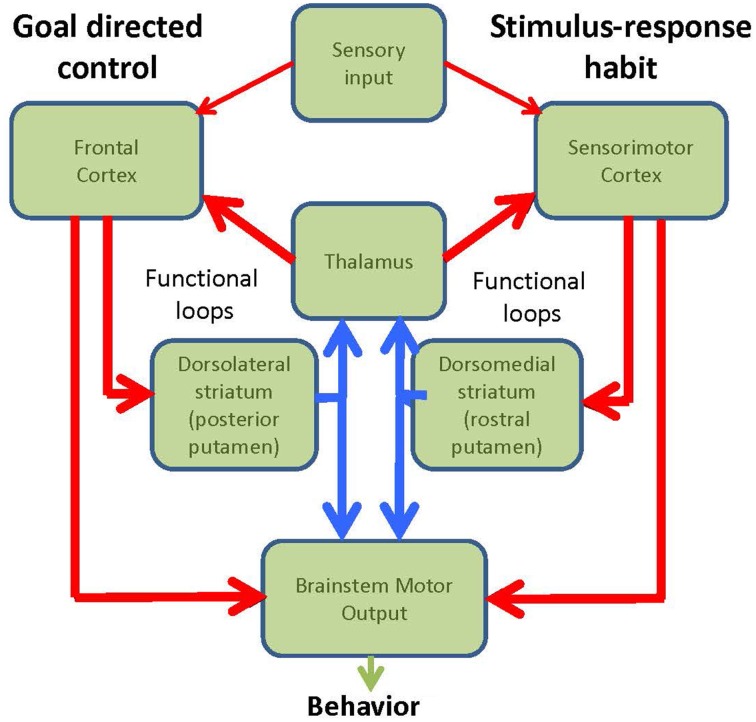
**Diagram of the functional loops involved in goal-directed and habitual behavior**.

## Functional anatomy of habitual vs. goal directed behavior

The nigro-striatal system has two major components, the associative loop and the motor loop. The associative loop comprises the dorso-medial SNpc that projects to the head of the caudate and rostral putamen, regions that have been associated with goal-directed behavior and executive functions (Yin and Knowlton, 2006). On the other hand, the ventro-lateral SNpc projects to the posterior putamen, which engages the sensorimotor circuits and habitual performance (Jog et al., 1999; Haber et al., 2000; Packard and Knowlton, 2002; Redgrave et al., 2010).

As humans, much of our thinking in daily life is made possible by engaging automatic or habitual control, e.g., walking, typing, etc…In fact, depending on the predictability of what we are doing, we frequently switch back and forth between goal-directed to habitual control. Goal-directed cognition (e.g. listening, talking) is often performed simultaneously while carrying out predictable sensorimotor tasks under habitual control (e.g. making tea, driving). Social interactions are characterized by the need to perform simultaneous and sequential activities while attending to multiple stimuli. We suggest that multi-tasking in humans could be an important vulnerability factor that puts the ventro-lateral subpopulation of dopaminergic neurons at greater pathological risk. Our hypothesis is that switching in and out of habitual control requires an unusually demanding anatomo-physiological network, which makes the dopaminergic neurons servicing sensorimotor territories of the basal ganglia especially vulnerable. We will argue that this represents a key factor for the neuronal degeneration associated with PD. As the ventro-lateral SNpc/caudal putamen (habitual system) becomes dysfunctional in PD, the goal-directed system has to be recruited to perform the previously automatic habitual tasks. This compensatory mechanism could have a “double-sword” effect by shifting the vulnerability to the now overloaded dopaminergic neurons in dorso-medial SNpc. This process could further propagate the neurodegeneration (Halliday and McCann, 2010).

## Factors associated with SNpc degeneration

Several studies analyse and discuss the nigro-striatal features that have been associated with potential degeneration of dopaminergic SNpc cells in PD (Hirsch et al., 2013; Sulzer and Surmeier, 2013). However, most of the identified characteristics are shared with the more medially located DA cells that seem to be less vulnerable in PD. Here we summarize some of the more relevant anatomo-functional markers that have been suggested to confer vulnerability to nigro-striatal neurodegeneration, which is the preferential and initial site of significant neuronal death in PD. We acknowledge the major current interest in the evolution of synuclein deposits (typically giving rise to Lewy bodies) in different regions of the nervous system. However, the actual significance of such aggregates to explain symptoms and to cause neurodegeneration in PD is unsettled yet (van de Berg et al., 2012).

### Neuromelanin

Interestingly, mammals generally show few melanized dopaminergic cells, which become more prominent in primate species and are particularly abundant in the human mesencephalon (Herrero et al., 1993). However, the presence of neuromelanin *per se* seems not to account for the specific vulnerability pattern of ventro-lateral tier, because not all brainstem (or anywhere else) pigmented neurons die in early PD (Gibb, 1992; Fedorow et al., 2005).

### Mitochondrial stress

Dopaminergic neurons are under a high mitochondrial oxidant stress (Surmeier et al., 2011). In fact, calcium entry through L-type channels during autonomous spiking, which characterizes this population, increases the vulnerability of SNpc dopaminergic neurons to the toxins 6-OHDA and MPTP; substances that are used to create animal models of PD (Chan et al., 2007). Thus, reduced complex I mitochondrial activity and elevated oxidant stress (Guzman et al., 2009) is a likely important factor in the PD-related pathogenesis of SNpc (Schapira, 2008; Blesa et al., 2015).

### DA and vesicular transporters

The activity and distribution of the synaptic dopamine transport (DAT) protein represents an entry for neurotoxic substances such MPTP and 6-OHDA (Dauer and Przedborski, 2003). DAT shows a dorso-ventral gradient with the expression of higher levels of glycosylated (mature, highly functional) protein found in the vulnerable ventral SNpc (Reyes et al., 2013). However, these neurons did not show a uniform expression of glycosylated DAT (Reyes et al., 2013). Together with the fact that other midbrain areas show glycosylated DAT, makes it unclear how these small expression differences could account for the differential vulnerability observed between SNpc and VTA neurons or between dorsal and ventral SNpc DA neurons (Gonzalez-Hernandez et al., 2004). Alternatively, another possible vulnerability feature for these neurons is their decreased vesicular accumulation of DA57 (Liang et al., 1996; Damier et al., 1999) and reduced levels of VMAT2 (Pifl et al., 2014). Failure to store DA into pre-synaptic vesicles appropriately would lead to higher cytoplasm levels of free dopamine and formation of cytotoxic free radicals. However, the extent to which this factor is able to account for the regional vulnerability of ventral midbrain DA neurons remains to be established.

### Dopaminergic striatal axonal arborization

The nigro-striatal projection exhibits one of the highest levels of divergent arborization (Matsuda et al., 2009; Bolam and Pissadaki, 2012). Thus, it has been estimated that a DA neuron that terminates in the rodent dorsal striatum has 102.165–245.103 synapses, while the number of synapse associated with corresponding neurons from the VTA are in the range of 12.351–29.644 (Bolam and Pissadaki, 2012). Importantly, in humans, the increase in the numbers of DA neurons (12.000 in rats vs. 382.000 in humans) has not kept pace with the striatal volume that these neurons innervate *(Vol str in mm*^3^*: rats: 19.9*^30^*; humans: 6,280*^43^*)* (Bolam and Pissadaki, 2012). The inference is that the degree of divergence in humans must therefore be even greater, thereby creating a substantially greater metabolic and proteostatic load on the human DA cells that innervate the sensorimotor striatum (Dryanovski et al., 2013). It remains to be ascertained if the latero-medial gradient of neuronal loss exhibited in the SNpc in PD is paralleled by degree of arborization.

### PD etiopathogenesis is multifactorial

While the differential features summarized above may play a significant role in nigro-striatal neurodegeneration, current evidence strongly indicates a multi-factorial origin for PD. Thus, mutation of single genes (i.e. parkin, LRRK-2, DJ-1, synuclein) can lead to DA neuronal loss (with or without Lewy body aggregates), glucocerebrosidase (GBA) expression correlates not only with the risk of developing PD (Beavan and Schapira, 2013) but also with its progression (Brockmann et al., 2015). Moreover, several genetic loci have been associated with increased risk of developing PD (Nalls et al., 2014). Finally, several environmental, life-style habits and toxic exposure have also been associated with higher or lesser risk of developing PD (Ross and Abbott, 2014; Tanner et al., 2014; Tanner and Comella, 2015). Accordingly, it is unlikely that the origin of neurodegeneration in PD could be tight to a single pathogenic mechanism or event.

Here, we would like to stress some clinical observations. Thus, that at the onset of PD the neuronal cell loss is highly asymmetrical, and mainly affecting one unilateral sub-group of DA neurons that innervate sensorimotor territories of the caudal putamen. As a consequence, the motor deficit is typically restricted to just one body part. These specificities are difficult to explain by changes in cellular markers that are widespread and feature in a high proportion of neurons.

A more integrative explanation that might bridge the gap between specific molecular abnormalities and the selective vulnerability of SNpc is to consider the functional anatomy of the nigro-striatal system as a significant risk factor. Our suggestion is that the early loss of ventro-lateral tier neurons could be determined by their role in the acquisition and control of automatic movements, and the resultant switching between goal-directed and habitual modes. This suggestion is supported by recent data where it has been reported that DA neurons are engaged by multiple events in tasks that have cognitive and sensorimotor components (Matsumoto and Takada, 2013), in addition to events associated with reward prediction (Schultz et al., 1997). It seems then, that DA neurons can be subdivided into functionally separate subpopulations; the ones specially coding reward prediction errors that are concentrated medially in the VTA, and neurons located in lateral SNpc that are responsive to a much wider range of salient sensory events, including to those associated with reward (Matsumoto and Hikosaka, 2009; Matsumoto and Takada, 2013). In rodents, these data are supported by observations that SNpc neurons also signal the initiation or termination of self-paced sequential behavior (Jin and Costa, 2010). This start/stop related activity emerged with learning, was specific for particular actions, and did not reflect timing or movement speed related actions. These data further support our hypothesis that, ventro-lateral DA neurons are sensitive to and activated by numerous aspects of action performance, learning and task switching. This more frequent pattern of activation of lateral SNpc neurons could represent an additional if not fundamental metabolic load that, associated with the features summarized above, could confer specific vulnerability to these cells.

## Parkinson's disease as a consequence of human behavior

Early in life, humans undergo an extended period of learning, during which a wide range sensorimotor, perceptual, cognitive and social skills, are acquired. Many of these skills have repetitive components, so after the first few decades, much of our behavior contains embedded fragments of automatic stimulus-response control. Many of these naturalistic automatic ‘chunks’ would, in a laboratory setting, turn out to satisfy formal criteria for stimulus-response habits—comparative insensitivity to outcome devaluation (Adams and Dickinson, 1981). An important consequence of establishing automatic habits is that it affords the possibility of undertaking multiple tasks simultaneously. These may consist of two or more automatic actions (walking and chewing gum), or a combination of an automatic and a goal directed action (walking and talking).

Becoming bipedal, thereby freeing the upper extremities to manipulate, allowed independent cognitive functions while doing things on the move. Consequently, the motor system in general, and the cortico-basal ganglia loops in particular, adapted to accommodate parallel processing. Dopaminergic activity, with its ability to modulate striatal excitability, plays an essential role in the initiation and expressions of self-paced stimulus-response. Accordingly, the dopaminergic nigro-striatal system becomes under increasing demand, especially the portion regulating habitual behavior. As a result, we believe, PD is the pathological result of increased functional demand on the nigro-striatal system produced by the evolutionary imperative for multitasking. Until recently this increased load had no apparent negative consequences, perhaps because life expectancy was short and below the risk age for developing PD. However, this has changed drastically, and the anticipated increase in future life expectancy is likely only to exacerbate this situation.

Thinking ahead, we realize that an ultimate research goal would be to replicate or simulate comparable levels of multitasking in suitable animal models to test the proposed hypothesis. This will pose a significant experimental challenge. However, our specific aim will be to establish non-human primate and rodent models in which ventro-lateral dopaminergic neurons are put under long-term functional stress.

## Conflict of interest statement

The authors declare that the research was conducted in the absence of any commercial or financial relationships that could be construed as a potential conflict of interest.
