# Corrigendum: Habitual Behavior and Dopamine Cell Vulnerability in Parkinson Disease

**DOI:** 10.3389/fnana.2015.00146

**Published:** 2015-11-17

**Authors:** Ledia F. Hernández, Peter Redgrave, José A. Obeso

**Affiliations:** ^1^Centre for Integrative Neuroscience A.C., Fundacion HM, Hospital HM Puerta del Sur, Mostoles and CEU San Pablo UniversityMadrid, Spain; ^2^Center for Networked Biomedical Research on Neurodegenerative Diseases, Institute Carlos IIIMadrid, Spain; ^3^Department of Psychology, University of SheffieldSheffield, UK

**Keywords:** Parkinson disease, habitual and goal-directed behavior, vulnerability, dopamine, substantia nigra pars compacta

Figure 1 of the article by Hernandez et al. (2015) contained a minor error, which we hereby rectify. In the original figure dorsolateral striatum (posterior putamen) is shown as part of the goal-directed loop while dorsomedial striatum (rostral putamen) is shown as part of the habitual loop. These boxes are switched. We therefore re-submit Figure 1 with the correct boxes in the middle panel.

**Figure 1:**
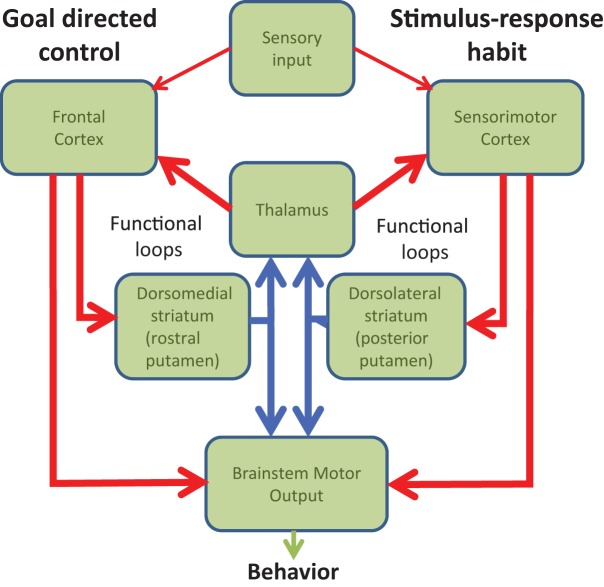
**Diagram of the functional loops involved in goal-directed and habitual behavior**.

## Conflict of interest statement

The authors declare that the research was conducted in the absence of any commercial or financial relationships that could be construed as a potential conflict of interest.

